# Extracellular vesicles and cancer stem cells: a deadly duo in tumor progression

**DOI:** 10.3389/or.2024.1411736

**Published:** 2024-07-18

**Authors:** Akram Tayanloo-Beik, Azin Eslami, Masoumeh Sarvari, Hasan Jalaeikhoo, Mohsen Rajaeinejad, Mohsen Nikandish, Ali Faridfar, Mostafa Rezaei-Tavirani, Ahmad Rezazadeh Mafi, Bagher Larijani, Babak Arjmand

**Affiliations:** ^1^ Cell Therapy and Regenerative Medicine Research Center, Endocrinology and Metabolism Molecular-Cellular Sciences Institute, Tehran University of Medical Sciences, Tehran, Iran; ^2^ Iranian Cancer Control Center (MACSA), Tehran, Iran; ^3^ AJA Cancer Epidemiology Research and Treatment Center (AJA-CERTC), AJA University of Medical Sciences, Tehran, Iran; ^4^ Student Research Committee, Aja University of medical sciences, Tehran, Iran; ^5^ Proteomics Research Center, Shahid Beheshti University of Medical Sciences, Tehran, Iran; ^6^ Department of Radiation Oncology, Imam Hossein Hospital, Shaheed Beheshti Medical University, Tehran, Iran; ^7^ Endocrinology and Metabolism Research Center, Endocrinology and Metabolism Clinical Sciences Institute, Tehran University of Medical sciences, Tehran, Iran

**Keywords:** cancer stem cells, extracellular vesicles, tumor microenvironment, drug resistance, precision medicine

## Abstract

The global incidence of cancer is increasing, with estimates suggesting that there will be 26 million new cases and 17 million deaths per year by 2030. Cancer stem cells (CSCs) and extracellular vesicles (EVs) are key to the resistance and advancement of cancer. They play a crucial role in tumor dynamics and resistance to therapy. CSCs, initially discovered in acute myeloid leukemia, are well-known for their involvement in tumor initiation, progression, and relapse, mostly because of their distinct characteristics, such as resistance to drugs and the ability to self-renew. EVs, which include exosomes, microvesicles, and apoptotic bodies, play a vital role in facilitating communication between cells within the tumor microenvironment (TME). They have a significant impact on cellular behaviors and contribute to genetic and epigenetic changes. This paper analyzes the mutually beneficial association between CSCs and EVs, emphasizing their role in promoting tumor spread and developing resistance mechanisms. This review aims to investigate the interaction between these entities in order to discover new approaches for attacking the complex machinery of cancer cells. It highlights the significance of CSCs and EVs as crucial targets in the advancement of novel cancer treatments, which helps stimulate additional research, promote progress in ideas for cancer treatment, and provide renewed optimism in the effort to reduce the burden of cancer.

## Introduction

Globally, cancer is considered one of the leading causes of death. It is estimated that there will be almost 26 million new cancer cases and 17 million deaths per year by 2030 [1]. The tumor microenvironment (TME) consists of various groups of cells with different characteristics and differential stages. Cancer stem cells (CSCs) are a small, heterogeneous subpopulation of cancer cells in most tumors. CSCs are responsible for most of the challenges encountered in cancer management. CSCs were first recognized and investigated in acute myeloid leukemia (AML) [2]. In accordance with their characteristics, including stemness and self-renewability, CSCs play an important role in tumor initiation, progression, drug resistance, relapse, and metastasis [3]. It is assumed that CSCs may originate from normal stem cells or progenitor cells, which justifies their specific characteristics [4]. However, a body of evidence has proposed that CSCs may result from stem-program activation and dedifferentiation of other tumor cells. Moreover, CSCs could evade the immune response by promoting M2 macrophage polarization and inhibiting the T-cell response [5]. The carcinogenic properties of CSCs are a direct result of a number of mechanisms, some of which are known. The mammalian target of rapamycin (mTOR) is an example of the signaling pathways responsible for the survival and pathogenesis of CSCs. Medulloblastoma CSCs have an overactivated mTOR pathway. These signaling pathways can be considered appropriate therapeutic targets, and the inhibition of signaling pathways in medulloblastoma cancer has led to increased sensitivity to radiotherapy and better results. Rapamycin is one of the drugs interfering with this signaling pathway [6, 7]. The Wnt pathway is another molecular mechanism that is responsible for the regulation of proliferation, differentiation, adhesion, migration, and self-renewal of CSCs [8, 9]. The significance of the Wnt pathway in cancer progression has made the designation of inhibitory drugs inevitable. The latest clinical trial on this matter has been the use of Wnt inhibiting factor 1 (WIF1) in prostate cancer. WIF1 has been found to increase sensitivity of prostate cancer patients to paclitaxel and etoposide [10]. NOTCH pathway activation has also been reported to play a role in cancer activation and metastasis, as well as the indication of drug resistance in carcinogenic tissues. This signaling mechanism is also favorable for a therapeutic approach. Research indicates that the use of NOTCH3 inhibitory agents can enhance the effectiveness of doxorubicin in treating hepatocellular carcinoma [11, 12]. CSCs have the ability to upregulate drug efflux transporters that protect them from chemotherapy damage [5]. Tight regulation of reactive oxygen species (ROS) and an increased ability to repair DNA damage are other protection mechanisms of CSCs [13]. Therefore, it is supposed that CSCs, which are commonly resistant to cancer therapy, reproduce the tumor cells again after the main tumor bulk shrinkage, hence leading to an unavoidable relapse [14]. Extracellular vehicles (EVs), or bilayer vesicles, of different types and sizes, are released from various cells, both healthy and cancer cells, including exosomes (50–100 nm), microvesicles (MVs) (100–1,000 nm), and apoptotic bodies (400–1,000 nm). Exosomes originate from the endosomal system, although MVs originate from outward blebbing of the plasma membrane. Apoptotic bodies are produced by the apoptosis process. EVs have diverse bioactive contents, such as long non-coding RNAs (lncRNAs) and micro-RNAs [15]. EVs mediate crosstalk between cells and the TME. EVs and other environmental factors determine the biological behavior of cells, including growth, differentiation, immune response, migration, and metastasis. By virtue of their contents, EVs could lead to genetic and epigenetic alterations that determine the cells’ fate in the TME. For instance, EVs derived from aggressive cancer cells could transfer aggressiveness and invasiveness characteristics to recipient cells. Moreover, it is hypothesized that communication between the primary tumor and metastasis is achieved through EVs. Additionally, EVs are responsible for increasing cancer cells’ stemness through tumor sphere formation [16]. Cancer progression is provoked through multiple pathways. PI3K/AKT and MAPK/ERK signaling pathways are responsible for tumorigenesis of EVs, according to a study conducted on gastric cancer patients [17]. Moreover, tumor-derived EVs have shown a capacity to activate neutrophils and aid in the remodeling of a proper tumor microenvironment [18]. This feature makes EVs a great therapeutic target in future studies [19]. Furthermore, this signaling pathway is capable of inducing drug resistance in malignant tissues. Thus, its inhibition culminates in a better response to conventional chemotherapy agents. An example to describe the above matter is the increased sensitivity of malignant breast cancer cells to trastuzumab. This increased sensitivity is the result of the depletion of HER 2-enriched EVs in breast cancer patients [20, 21]. EVs and CSCs could be identified and isolated through various methods (ultracentrifugation, ultrafiltration, and flow cytometry), including their cell surface markers and and specific CD markers [13]. Furthermore, discovering the exact mechanisms and associations between CSCs and EVs, their influence on different stages of tumor growth, and mechanisms of resistance to conventional radiation and chemotherapy is important. Additionally, bioengineering of the EVs by altering their contents and surface markers, changing their pathways and tropism to tissues, and proposing promising treatments is essential. In this study, we review and discuss the characteristics of CSCs and EVs, their clinical implications in the pathophysiology of cancer, and novel therapeutic targets to shed light on this area.

## Extracellular vesicles and cancer stem cells: emerging players in tumor progression

### Extracellular vesicles

**FIGURE 1:**
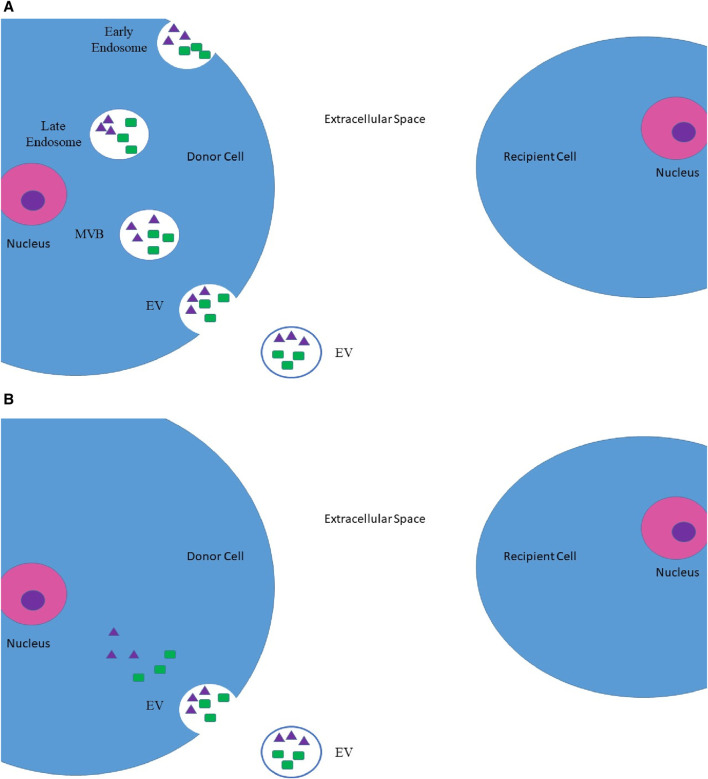
Origins of EVs. **(A)** Inward budding of an endosome, which later on transforms into a MVB. This MVB may eventually be secreted into the extracellular space as EV. **(B)** Direct budding of EV from the plasma membrane. These EVs may contain protein molecules (such as oncopeptides and oncoproteins), which are represented by green rectangles, and nucleic acids (such as DNA segments and RNA segments (including mRNAs, microRNAs, and lncRNAs)), which are represented by purple triangles [23]. *MVBs, multivesicular bodies; EV, extracellular vesicle; lncRNAs, long non-coding RNAs.

Pan and Johnstone first described EVs. They are defined as membrane-originated vesicles stemming from either the endosomal system or the plasma membrane itself [22, 23]. Once EVs were discovered, we needed to classify them. One method of vesicle characterization is to consider their size. Xu et al. proposed a rather simplified classification regarding the size of these vesicles. Table 1 further illustrates the classification [24]. Another method to aid in further classification of EVs is to consider their source of origin. Figure 1 is a visual aid for these classifications [23].

**TABLE 1:** EVs are classified based on their size [24].

Classification	Size (nm)	Protein content	Surface marker	Origin
Small EVs	50–120	Collagen alpha-1, MAM domain-containing protein 2, EGF-containing fibulin-like extracellular matrix protein 2, and protein disulfide-isomerase A4 [25]	Tetraspanins, CD9, CD63, and CD81 [26, 27]	Endosomes [27] of blood cells, central nervous system, dendritic cells, adipocytes, mast cells, endothelial cells, cardio myocytes, hepatocytes, and intestinal cells [27, 28]
Intermediate EVs	200–300	Protein disulphide-isomerase A6, 3-ketoacyl-CoA thiolase A, and peroxisomal [25]	ARF6 and VAMP3 [29] (mainly in micro vesicles)	Plasma membrane of different cells [29]
Large EVs	>500	Stress-70 protein, heat shock protein, gelsolin, myosin, annexin A1, 14-3-3 protein zeta/delta and alpha, moesin, guanine nucleotide-binding protein G, etc. [25]	TSP, C3b [29] (mainly in apoptotic bodies)	Plasma membrane of different cells [29, 30]

^a^ EVs, extracellular vesicles; nm, nanometer.

**FIGURE 2:**
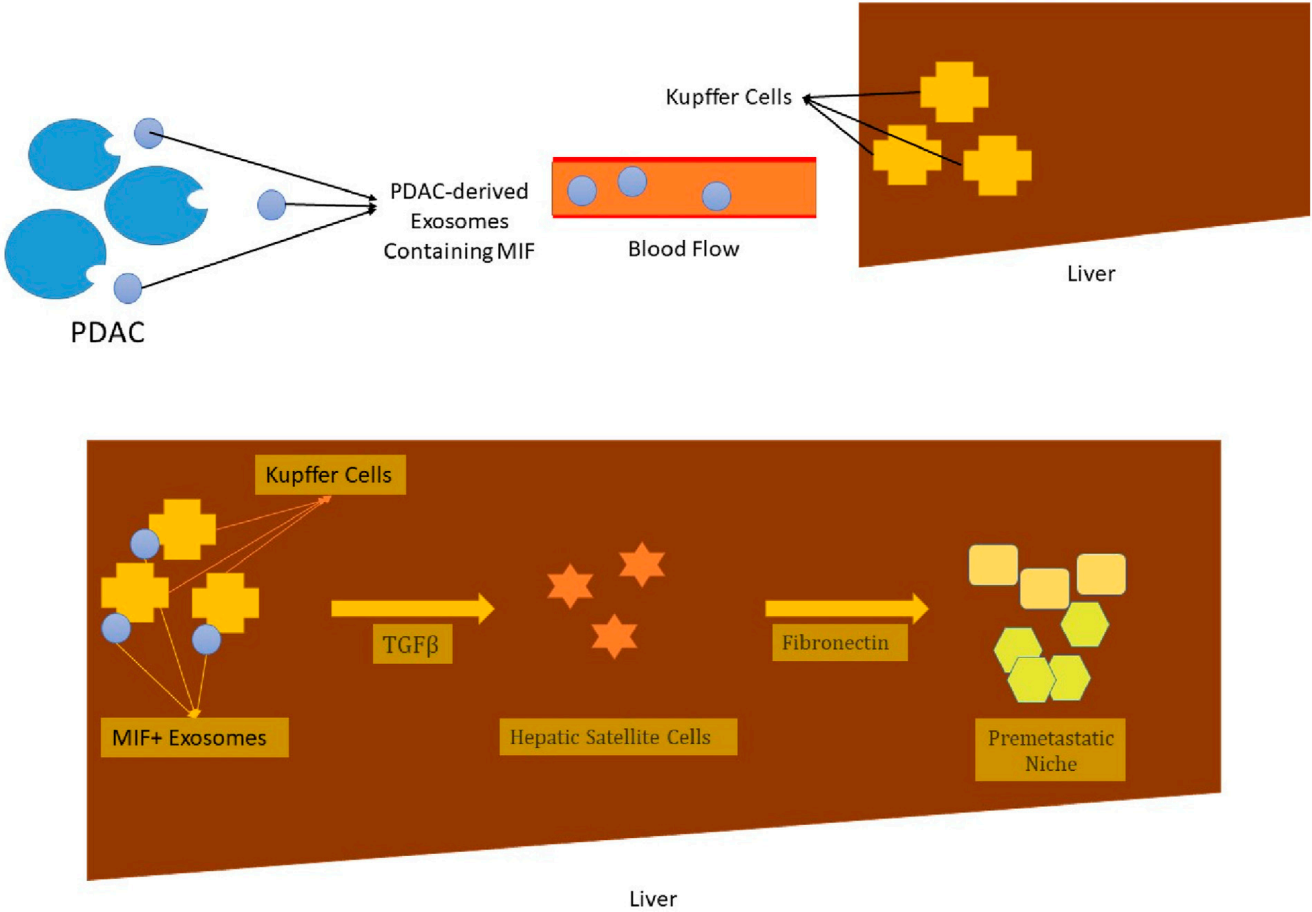
The exosomes, which are derived from PDAC malignant cells, are captured by liver macrophages (Kupffer cells), after being transported to their site of action via the blood flow. These exosomes contain MIF, which leads to higher production of TGF-β. Under the direct influence of this chemokine, hepatic satellite cells secrete fibronectin molecules. The provoked immigration of bone marrow cells such as neutrophils and macrophages and their trapping in the fibronectin net leads to the formation of a pre-metastatic niche. The liver metastasis of PDAC is the ultimate outcome of it all [35]. * PDAC, pancreatic duct adenocarcinoma; MIF, macrophage migration inhibitory factor; TGF-β, transforming growth factor β.

EVs may also be categorized based on their content. These membrane-limited particles are capable of carrying nucleic acids and protein molecules. The transfer of such contents between the donor and recipient cells culminates in the transformation of the recipient cell’s characteristics and complicated intercellular communication, both of which may lead to physiological or pathological outcomes [31]. These intercellular communications can play multiple simultaneous roles in tumor pathology. For instance, tumor-originated EVs, also known as oncosomes, establish intercellular communication between malignant cells and their surrounding stromal cells and the microenvironment. The outcome of such communication is the transformation of the recipient’s characteristics and the establishment of a tumor-promoting niche. The processes of angiogenesis and immunosuppression will be accordingly carried out in this setting [27, 32, 33]. Moreover, the interplay and intercommunication of tumor cells and their microenvironment and the promoted vascular leakiness triggered through the secreted exosomes result in the formation of the pre-metastatic niche. Such niches can adversely affect tumor progression, invasion, and metastasis [31, 34]. Pancreatic ductal adenocarcinoma (PDAC)-derived exosomes are a tangible instance of the influential role of exosomes in cancer metastasis. As these exosomes are accumulated by liver macrophages, they lead to an increase in levels of macrophage migration inhibitory factor (MIF) and ultimately a stimulation of malignant cells. The elimination of such exosomes halted liver metastasis in PDAC rats [35] (Figure 2).

EVs directly contribute not only to tumor progression and metastasis but also to therapeutic resistance. EVs are reportedly able to expel intracellularly accumulated chemotherapy agents such as cisplatin [36] and doxorubicin [37]. This characteristic is transferrable from drug-resistant cells to drug-sensitive cells. The transport of P-gp and nucleic acids leads to such a phenomenon and promotes drug resistance [38, 39]. The superiority of EVs in these mechanisms can stem from various grounds. To start with, EVs are capable of transporting chemicals and proteins and nucleic acids, DNA, and RNA fractions. Since EVs originate from the plasma membrane, they carry receptors (such as growth factor receptors) and transmit them to the recipient cells. EVs tend to have degrading enzymes, which provide protection for their contents. The protected EV contents are able to travel further distances [40].

With all these in mind, one may interpret that EVs are valuable targets in therapeutic procedures. This assumption is theoretically significant. EVs are to be employed in defeating cancer in two independent ways. One obvious way is intervention by cancer-derived EVs in order to prevent cancer-promoting pathways. For instance, it is desirable to cease the production or release of EVs by their cells of origin, remove them from the bloodstream and tissue, or interfere with their uptake by their target cells [41]. Fabbri et al. proposed that GW4869, an n-SMase inhibitor, works as a neutral sphingomyelinase inhibitor to stop the release of EVs. Treatment with this drug causes lower production of malignant cells in mice with Lewis lung carcinoma [19, 41–43]. Another possible route of action for the implications of EVs in cancer therapy is to consider them as a means of transportation for engineered drugs and molecules. Despite the lack of carefully tailored mechanisms for use of EVs, since 2005, trials have been launched to apply EVs as medical transporters. One of the first trials in this field involved the intradermal and intravenous injection of EVs, which were previously loaded with desirable antigens. These dendritic cell-derived EVs were administered to non-small cell lung cancer and metastatic melanoma patients. Although the therapeutic outcome of such trials has not been enormous, they still trigger the need to discover new realms of possibility [44–46]. To do so, another clinical trial was carried out in 2007. The administration of granulocyte–macrophage colony-stimulating factor from the patient’s own ascites via engineered EVs presented promising outcomes and cytotoxic T lymphocyte activation in colorectal cancer cases [47]. The use of EVs in breast cancer patients has similarly enhanced cytotoxic T-cell activity [48]. Furthermore, EVs, which were enriched with doxorubicin and paclitaxel, demonstrated higher mobility across the brain–blood barrier. Therefore, patients with brain cancer seem to benefit from this therapy modification [49, 50].

### CSCs

Another state-of-the-art discovery in the field of cancer is cancer stem cells (CSCs). Four decades ago, researchers introduced CSCs, a specific yet limited number of malignant cells that ensure the renewal and survival of the tumoral tissue [51, 52]. CSCs can be practically isolated from a variety of solid and liquid cancers (including breast cancer [53], brain tumors (prolactinoma [54], glioblastoma [55], etc.), prostate cancer [56], lung cancer [57], liver cancer [58], colorectal cancer [59], and skin cancer [60]). However, they are not as easily attainable from other types of malignancies. Furthermore, there is a theory that suggests certain cells possess the ability to oscillate between being a CSC and not being one. The defining feature of such cells is that they are similar to normal tissue stem cells in the case of renewal and repopulation [61]. These cells harbor malignant niches. However, the identification, isolation, and eradication of such cells are not as feasible as one may hope [51]. A variety of studies highlight the significance of CSCs in tumor progression [62–64]. A set of transcriptional factors (including OCT4, SOX2, KLF4, MYC, NANOG, Wnt/TCF, STAT 3, and NF-κB), nucleic acid alterations (including RNA methylation, RNA splicing, and DNA methylation), and epigenetics are accountable for this phenomenon [65, 66]. Moreover, metastasis is also greatly provoked and facilitated by CSCs [67–69]. Knocking out CD133+ and CXCR4+ CSCs in pancreatic malignant tissue ceases tumor metastasis, leaving the tumorigenesis mechanisms untouched [69]. As CSCs abandon the primary tumor site, an epithelial–mesenchymal transformation (EMT) takes place. Mesenchymal cells are apt to have more facilitated mobility, a feature acquired by CSCs. The root of such transformation is an alteration in the transcriptional program [70], which is led by a network of stem cells. At the desired destination, the modifications reverse, and CSCs induce tumorigenesis at the second site [71]. Alongside their individual roles in cancer pathology, CSCs and EVs are identified as responsible for tumor progression and metastasis. There are several methods by which the abovementioned can be accomplished. For instance, colorectal CD133^+^ CSCs secrete exosomes that contain circ-ABCC. These exosomes induce stem-like features in non-CSCs and thus tend to multiply the population of CSCs in colorectal cancer patients [72]. Enhanced tumorigenesis is detected in esophageal cancer through CSC-derived exosomes containing *FMR1-AS1* [73]. In the same manner that has been previously discussed, the induction of glioblastoma CSCs is facilitated by exosomes, which deliver NOTCH1 protein and activate the related signaling mechanism [74]. Researchers have concluded that exosomes, which contain marker-related proteins, significantly contribute to the induction of stem-like properties in non-CSCs. Furthermore, proteins wrapping these particular CSC-derived exosomes are also responsible for the undesired consequences [74]. Not only are the CSC-derived exosomes accountable for tumor progression and tumorigenesis, but they are also amenable to triggering tumor metastasis. This phenomenon has been closely studied in several cases [75–78]. Even so, the precise mechanisms have not yet been described. Wang et al. conducted a study on clear cell renal cell carcinoma (CCRCC) and showed that exosomes secreted by CSCs are very helpful in starting and keeping EMT going. This is how pre-metastatic niches are assembled and tumor metastasis is promoted [79]. CCRCC is not the sole malignancy that has been closely investigated. The CSC-derived exosomes in lung cancer tissues have also been critically involved in the stimulation of metastasis [80]. Thyroid CSC-derived exosomes have similarly been transported to distant locations by carefully employing their miRNAs, lncRNAs, and proteins [81]. As mentioned previously, a thorough and detailed description of the mechanisms responsible is not yet available.

This is the reason why the number of ongoing clinical trials on possible suitable drugs for interfering with this crucial cell line in malignant cancers is not as large as one may desire. Although Hexum et al. managed to introduce several bicyclic cyclohexenones, these chemical agents target the NK-κB signaling pathway. As the signaling pathway is altered, the production of survival factors in CSCs diminishes. This method is actively tested in lung adenocarcinoma, prostate cancer, and T-cell lymphoblast cancer [82]. Moreover, the inhibition of hypoxia-inducible factor 1α (HIF-1α) by chemical agents such as LY294002 and rapamycin has shown promising effects in eradicating CSC niches [83, 84]. For the time being, CSCs are rather enigmatic for researchers. Further exploration into the novel concept may help discover possible diagnostic, prognostic, or therapeutic targets in the near future.

### CSC-derived EVs

EVs are likely to stem from a normal cell and a cancer cell or a CSC. These EVs may bear differences in their cargo, although they have basic resemblances. An endosomal sorting complex normally forms EVs. Moreover, they tend to halt molecules based on their physiological state at the time of production [85]. Once these vesicles arrive at their target cells, they have two ways of communicating: 1) they may directly bind to the proteins and lipid ligands of the plasma membrane of the target cells and thus activate a specific signaling pathway; 2) they may fuse to the target cell and transfer their cargo directly into the target cell [86]. These vesicles play numerous roles in the human body, critically contributing to the formation of inflammatory responses. This occurs in physiological states, such as developing an innate immune system against infections [87], and in pathological conditions such as rheumatoid arthritis [88], type 2 diabetes [89], and other autoimmune diseases [90, 91].

Malignant cells may produce several EVs. A distinct number of studies have been dedicated to investigating the mechanisms through which tumor-derived EVs function. Sun et al. carried out one such study. This study reveals high levels of miRNA-21 in the CD133+ cells of glioblastoma cell lines. It has been proposed that the miRNA-21/VEGF pathway enhances angiogenesis in these malignant conditions [92]. A study on clear renal cell carcinoma revealed high levels of miRNA-19b-3p in the exosomes derived from CD105+ cells. This miRNA-induced cell migration increased the levels of PTEN protein [93]. CD105+ cells from renal carcinoma reportedly contain higher levels of 24 types of miRNA and lower levels of 33 types of miRNA. This miRNA regulation facilitates tumor growth and tumor invasion [94, 95]. According to Domenis et al., high levels of miR-19b-3p are detectable in glioblastoma CSC-derived EVs. The increase in miR-19b-3p levels elevates tumor metastasis [96]. Colorectal cancers have also been the target of studies. It has been depicted that an increase in the levels of triphosphate RNAs in CSC-derived EVs in colorectal cancer is responsible for the formation of an immunosuppressive environment that protects malignant cells against natural defense mechanisms [97]. Ordinary EVs do not contain the same products at the same level [98]. In cases where they do contain the same material, different signaling pathways are activated, leading to different clinical results [30, 99]. Table 2 summarizes the functions, origins, and cargo of CSC-derived EVs.

**TABLE 2:** CSC-derived EV functions, origins, and cargo.

EV cargo	Cancer cell	Function	Reference
miR-19b-3p	Glioblastoma	Tumor metastasis	[96]
Increase in levels of 24 miRNAs and decrease in levels of 33 miRNAs	CD105+ cells of renal carcinoma	Facilitated tumor growth and tumor invasion	[94, 95]
miRNA-19b-3p	Clear renal cell carcinoma	Cell migration	[93]
miRNA-21	Glioblastoma	Angiogenesis	[92]

## Extracellular vesicles and cancer stem cells in tumor heterogeneity

### Influence on tumor subpopulations and clonal evolution

Various theories explain the tumor’s characteristics. It is hypothesized that, usually, a group of cells during the treatment escapes therapies and remains in the body. These residual cells, through clonal evolution, could change in the tumor environment and produce novel sub-clones. These novel sub-clones could inherit new properties, including self-renewability, pluripotency, invasion, and migration, through genetic and epigenetic alterations. Based on CSC theory, CSCs, as sub-clonal populations, play a major role in relapses and metastases [100]. EVs, secreted from immune, normal, and cancer cells in the tumor environment, play a pivotal role in these alterations. Additionally, EVs contain various bioactive molecules, including proteins, lipids, and nucleic acids, which are released into the extracellular environment and transfer bioactive material to neighboring or distant cells. In accordance with the nucleic acid material of EVs, lncRNAs and micro-RNAs, as the most common types of non-coding small RNAs (ncRNAs), contribute to tumor signaling pathways (Table 3). In this regard, they control the oncogenic cellular pathways through genetic and epigenetic alterations at the translational and posttranscriptional levels. In this regard, angiogenesis is a key factor in tumor progression. Specific micro-RNA expression patterns through altered endothelial cell pathways help in this process. Accordingly, micro-RNA-16, micro-RNA-21, micro-RNA-23a, micro-RNA-29, micro-RNA-100, micro-RNA-221, and micro-RNA-222 are known to participate in vascular progression [131]. For instance, a body of literature has shown that CSC-EVs affect the key regulatory pathways, including TGF-β, NF-kB, protein kinase B, Wnt/β-catenin, and NOTCH. EVs increase TGF-β, NF-kB, and protein kinase B levels, which alters the immune response [16]. Consequently, this immune response modulation provides a suitable environment to survive the CSCs [16]. For instance, Cheng et al. investigated the colorectal CSCs and related EVs. Micro-RNA-146a-5p is considered a major component of colorectal CSC-related EVs, which act through the Numb pathway to enhance tumorigenicity in the target cells. Furthermore, these EVs increase tumor-filtrating CD66^+^ neutrophils and decrease tumor-infiltrating CD8^+^ T cells, thereby manipulating the immune response [124]. On the other hand, EVs derived from immune cells, including CD8^+^ T cells, help eradicate tumor cells. Micro-RNA-298–5p, as one of the contents of the CD8^+^ T cell-derived EVs, activates caspase-3. This activation induces apoptosis in mesenchymal stem cells (MSCs) and reduces CSCs in the tumor environment. In this regard, micro-RNA-23a-3p, derived from the endoplasmic reticulum of hepatocellular carcinoma (HCC) cells, inhibits T-cell function. Micro-RNA-23a-3p reduces T-cell activation by targeting PTEN in macrophages [132, 133]. CSC-derived EVs are considered a promising tool in cancer therapy. An *in vivo* study on mouse models has investigated the therapeutic effect of antibodies against the antigens on the surface of EVs. In breast cancer models, anti-CD9 or anti-CD63 antibodies diminished the rate of lung, lymph node, and thoracic cavity metastasis [134]. Interestingly, some particles are suggested as a promising tool for eliminating oncogenic CSC-derived EVs. For instance, hemopurifiers in patients with advanced and/or metastatic squamous cell carcinoma of the head and neck may clear immunosuppressive exosomes in combination with pembrolizumab [13]. Altogether, diverse EVs and their contents in communication with CSCs are the main players in the tumor environment and prognosis.

**TABLE 3:** Micro-RNAs in various cancers. Micro-RNAs, as a group of non-coding RNAs in extracellular vesicles, play an important role in tumor progression, survival, and metastasis. Unique micro-RNA expression profiling has been demonstrated for many types of cancer, including breast cancer, lung cancer, prostate cancer, and leukemia. Dysregulated (up- or downregulated) micro-RNA expression patterns can contribute to tumorigenesis by targeting oncogenes or tumor suppressor genes, promoting cell proliferation, invasion, and metastasis and inhibiting apoptosis.

Cancer	Micro-RNA
Breast cancer	micro-RNA-130a-3p [101], micro-RNA-600 [102], micro-RNA-638 [103], micro-RNA-590-5p [104], and micro-RNA-378a-3p [105]
Squamous cell carcinoma (SCC)	micro-RNA-495 [106] and micro-RNA‐142‐5p [107]
Cervical cancer	micro-RNA-145 [108]
Ovarian cancer	micro-RNA-328–3p [109]
Pancreas cancer	micro-RNA-146b-3p [110]
Hepatocellular carcinoma (HCC)	micro-RNA-206 [111], micro-RNA-375 [112], micro-RNA-192-5p [113], micro-RNA-106b-5p [114], and micro-RNA-124 [115]
Osteosarcoma	micro-RNA-155 [116] and micro-RNA-26a [117]
Colon cancer	micro-RNA-194 [118], micro-RNA-215 [119], micro-RNA-221 [120], micro-RNA-92a [121], micro-RNA-195-5p [122], micro-RNA-302c [123], and micro-RNA-146a-5p [124]
Gastric cancer	micro-RNA-196a-5p [125] and micro-RNA-7-5p [126]
Glioblastomas	micro-RNA-603 [127] and micro-RNA-223 [128]
Non-small cell lung cancer (NSCLC)	micro-RNA-221/222 [129] and micro-RNA-223-3p [130]

### Impact on therapy resistance and relapse

**FIGURE 3:**
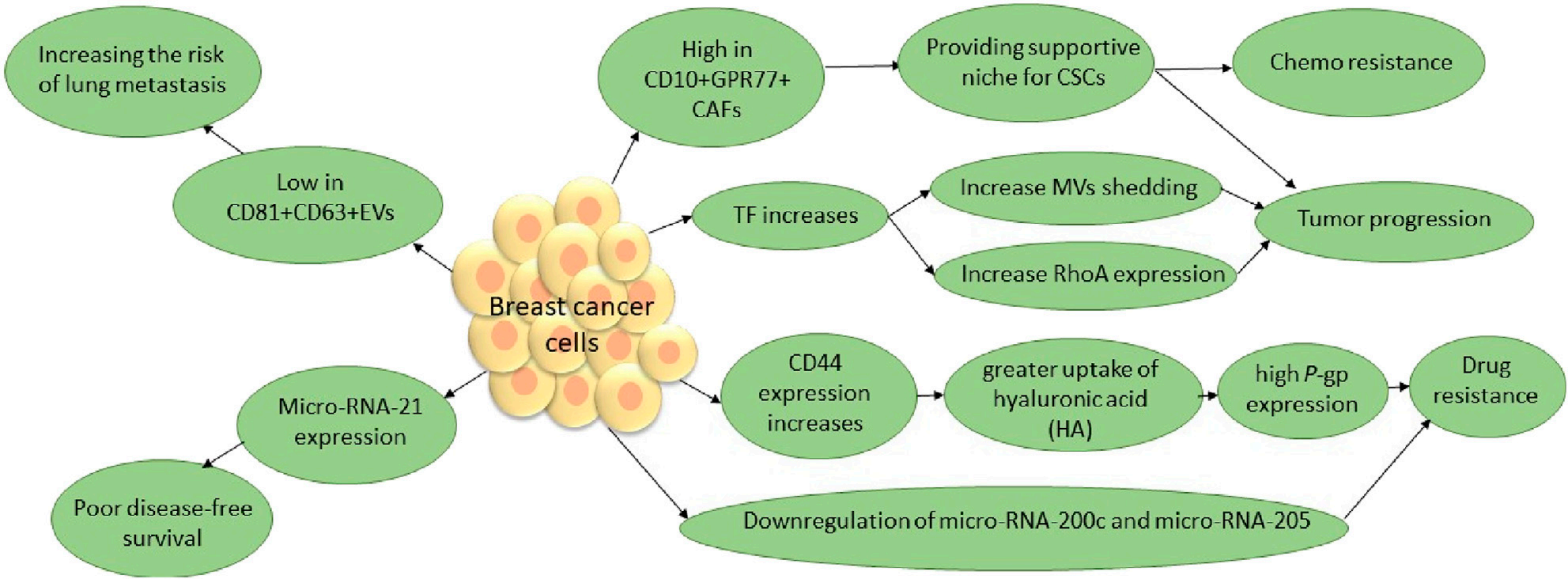
TF contributes to the release of MVs, a subgroup of EVs that play a pivotal role in tumors. RhoA, a small GTPase, has been identified as a key effector of cytoskeleton rearrangements and MV generation in tumor cells. Additionally, P-glycoprotein (P-gp), known as the multidrug resistance 1 (MDR1) protein, suggests that decreasing MVs by eliminating TF and RhoA, as well as P-gp in breast cancer, will improve susceptibility to anti-cancer therapeutics and tumor prognosis. A subset of CAFs in breast cancer, through surviving CSCs, leads to poor prognosis. In addition, evidence has shown that elevated levels of miR-21 expression are associated with aggressive disease status, including high tumor grade, negative hormone receptor status, and ductal carcinoma. The micro-RNA-200 family and micro-RNA-205 are generally known to suppress EMT, which leads to breast cancer progression [135, 139, 140].* CSCs, cancer stem cells; CAFs, cancer-associated fibroblasts; TF, tissue factor; MVs, micro vesicles; EMT, epithelial–mesenchymal transition.

A body of evidence has shown that EVs, through various pathways, including increasing anti-apoptotic characteristics, lead to carcinogenic cell survival [135–137]. MVs, as a sub-group of EVs, play an important role in tumor growth and aggressiveness. Cancer is one of the pathologies in which tissue factor (TF)-bearing MVs are increased. TF-bearing MVs are a main factor in tumor growth and aggressiveness [138] (Figure 3). A group of EVs are derived from immune cells and have diverse effects on tumors. For instance, dendritic cell (DC)-derived EVs participate in the activation of NK cells and improve the antigen-specific responses of CD4^+^ and CD8^+^ T cells. Researchers are investigating whether EVs from regulatory T cells suppress pathogenic Th1 responses through a miRNA-dependent pathway. Researchers also extensively investigate exosomes derived from tumor-associated macrophages (TAMs). A study stated that EV-derived TAMs contain two main micro-RNAs, namely, micro-RNA-29a-3p and micro-RNA-21-5p. These two micro-RNAs stimulate CD4^+^ T-cell differentiation into Th17 cells, inducing a higher regulatory T (Treg)/Th17 cell ratio [132, 133]. On the other hand, it is hypothesized that TAMs are similar to M2 macrophages, showing tumor-supportive phenotypes. It is well known that M2 macrophages, unlike M1 macrophages, play a significant role in tumor progression. The M1/M2 transition of macrophages is a dynamic process, and many factors are implicated in this transition. Meanwhile, EVs, as the main factor in macrophage polarization, provide a suitable microenvironment for tumor growth, angiogenesis, and metastasis [141]. Furthermore, Fathi et al. investigated a methodology that integrates EVs with cellular functions. They compared two metastatic and non-metastatic breast cell lines. They have seen that CD81^+^CD63+EV secretion from non-metastatic cell lines is more than that from metastatic lines. They showed that CD81^+^CD63+EVs increase the activity of immune cells that break down cells, increase the number of pro-inflammatory macrophages, and improve clinical outcomes. Additionally, they conducted a study on the function of CD81^+^CD63+EVs in melanoma cancer. In summary, they suggested that CD81^+^CD63+EVs contribute to restricting metastasis development in breast and melanoma in lung tissue and that tumors with low levels of CD81^+^CD63+EVs have a high tendency to develop lung metastasis [142]. A study investigated metastasis induction through CSCs in CCRCC and found that EVs are derived from CSCs in CCRCC patients with lung metastasis. These EVs apply their pro-metastatic properties and induce EMT through miR-19b-3p [79]. Therefore, targeting these CSCs and EVs, especially those that affect mesenchymal tumor stromal cells, will be useful in patients with refractory and metastatic cancers.

### Potential as targets for precision medicine

Recently, CSCs have been known as novel cancer therapy targets. Additionally, EVs, through their contents, determine the fate of cells by controlling cellular pathways. CSCs and related EVs play a critical role in drug resistance. Interestingly, EVs have the potential to be modified through their contents, including ncRNAs, which sheds light on novel anti-cancer therapies [143]. For instance, ALDH is known as a cell surface marker of CSCs in special tumors. ALDH helps leukemic CSCs escape the cytotoxic effects of ROS and, thus, chemotherapy. In this regard, pieces of literature have investigated the role of CD8^+^ T cells as a key player in tumor regression. They have shown that CD8^+^ T-cell exosomes could restrict the tumor stroma and MSCs. So this decrease in mesenchymal tumor stromal cells happened through a miRNA (miR-298-5p)-dependent pathway, not through pathways involving TNF-α or Fas [15]. A study by Seo et al. on EVs derived from CD8^+^ T cells suggested them as novel potential therapeutics, especially in metastatic refractory pancreatic cancer [132]. As we know, angiogenesis contributes to the progression of various malignant tumors. Micro-RNA-210 is overexpressed in hypoxic conditions in endothelial cells. Furthermore, this induction leads to capillary-like structures. On the other hand, overexpression of micro-RNA-221 and micro-RNA-222 has anti-angiogenic effects in endothelial cells. Hence, therapeutics that inhibit micro-RNA-210 and increase micro-RNAs, including micro-RNA-221 and micro-RNA-222, in endothelial cells through dysregulated angiogenesis help develop novel treatments [131]. MVs are capable of transferring oncogenic materials to adjuvant or distant tissues that are resistant to the tumor environment and circulation. Grange et al. tested the function of renal-CSC-derived MVs. They have shown that these CSC-derived MVs significantly trigger angiogenesis and metastatic spreading in the lungs [94]. Therefore, it is supposed that the best way to treat cancer is to eradicate MVs and inhibit their production. The release of MVs into the tumor environment depends on various factors, including the influx of calcium, TF, and hypoxia. TF, a transmembrane protein, contributes to tumor progression through MV production. Rondon et al. demonstrated that TF knockout in breast cancer cells may be helpful in anti-cancer therapy [135]. Hypoxia induces HIF production and expression of the Ras-related protein Rab-22A (RAB22A), which subsequently leads to MV production [140]. Furthermore, a study by Antonyak et al. has reported that GTPase RhoA silencing disturbs the MV synthesis pathway [144] (Figure 3). Therefore, novel anti-cancer therapies should specifically target EVs from their initial stage of production and their surface and surrounding environments.

## Extracellular vesicles and cancer stem cells in the tumor microenvironment

As previously discussed, EVs and CSCs are two major players in the process of cancer formation and metastasis. EVs have recently been held accountable for their role in cancer pathogenesis. They are responsible for tumor progression, metastasis, drug resistance, etc. [145]. This is the reason why EVs have become the focus of interest for both diagnostic and therapeutic approaches. Theoretically, the detection and calculation of circulating tumor-derived EVs could serve as an acceptable diagnostic or prognostic factor. These EVs and their surface markers, as well as their contents such as proteins and nucleic acids, can be considered valuable indicators of the malignant cell, which is their source of origin [146, 147]. Circulating EVs have been more beneficial in comparison to circulating malignant cells in terms of diagnosis [147]. This diagnostic tool has so far been applied to in several malignant conditions, such as ovarian, breast, and pancreatic cancer [148]. Furthermore, EVs are used in cancer therapy. We achieve this by targeting tumor-derived EVs, interfering with their function, and using EVs as a transportation tool to transport drugs into malignant tissues and cells [149]. The depletion of Her-2-positive MVs from the bloodstream in breast cancer patients is a tangible instance of targeting tumor-derived EVs [149]. Furthermore, we can use engineered EVs to deliver therapeutic agents like paclitaxel and lomustine to patients with somatonoma, providing a more precise and targeted therapy [150].

CSCs have recently been introduced as a limited subpopulation of malignant cells that bear inherent capabilities such as self-renewal, differentiation, and tumor induction. The asymmetrical division of CSCs culminates in the generation of at least two different cell populations: one group with self-renewal properties, responsible for tumor formation, and the other, a group of cells with differentiation capabilities [151].

These cell groups are detectable by their surface markers (CD44, CD24, and CD133). The prominent property of this subgroup of cells is their ability to induce tumor formation in distant locations. This phenomenon is the direct outcome of a network of signaling patterns, including microRNAs and Wnt/β-catenin, NOTCH, and Hedgehog signaling pathways [152].

CSCs’ unique characteristics have piqued interest and positioned them as potential future diagnostic and therapeutic targets. Detecting this subpopulation of malignant cells has made it possible to identify the existence of malignant tissues in one’s body. This method has been tested in patients with lung cancer [153, 154], as well as breast cancer [155]. The results have been promising. On the other hand, CSCs can serve as viable targets for therapy. There have been cases of head and neck cancer [156, 157], ovarian cancer [158, 159], and testicular cancer [160] that have designated inhibitor factors to deplete CSCs in patients [161].

The TME is defined as the complex of the extracellular matrix (ECM), recruited cells, and chemical factors that aid in harboring, preserving, and stimulating malignant cells [162]. Several studies have also demonstrated that the survival and renewal of CSCs heavily depend on the existence and maintenance of CSC niches [75, 163, 164]. These micro-environments are composed of the ECM, stromal and immune cells, chemicals (including growth factors and cytokines), and EVs. There is an ongoing endeavor to maintain the pH, hypoxia and angiogenesis, inflammation, and EMT constants. This ensures the safety and viability of the residing CSCs [165]. Both the TME and CSC niche are colonies consisting of a remarkable diversity of cell types: immune cells [including T lymphocytes, B lymphocytes, natural killer cells (NK cells) and natural killer T cells (NKT cells), M2 TAMs, myeloid-derived suppressor cells (MDSCs), dendritic cells (DCs), and tumor-associated neutrophils (TANs)], cancer-associated fibroblasts (CAFs), adipocytes, pericytes, and vascular and lymphatic endothelial cells [162, 166]. These components help provide a nurturing and nourishing environment for CSCs and malignant cells, which can, in turn, appear as a spectacular therapeutic aim. In this instance, Barone et al. introduced bevacizumab, a VEGF tyrosine kinase inhibitor, in glioblastoma. The resulting devastation of the perivascular niche and TME aids in increasing the survival time of glioblastoma patients [167]. This drug has also been tested for non-small cell lung cancer. Although in this trial a combination of bevacizumab and anti-hepatoma-derived growth factor was employed, the results were rather acceptable, and the CSC population decreased in terms of size [168]. Meanwhile, targeting the VEGF–VEGF2–NRP1 axis in glioblastoma patients has indicated promising results in diminishing CD133+ CSCs [169]. It is important to keep in mind that inhibiting growth factors will not necessarily lead to a depletion of CSCs. Reports indicate that the inhibition of growth factors in breast cancer has led to an increase in the number of CSCs. Hypoxia, resulting from a lack of angiogenesis, partially contributes to this [170, 171]. A thorough comprehension of the TME components and their mechanisms of action will aid researchers in designating the appropriate therapeutic approach.

### T lymphocytes

The intercellular connection between CSCs and their respective EVs and T lymphocytes is complicated. Theorists even theorize that CSCs may originate from T lymphocytes [172]. However, T lymphocytes continue to be the preferred cell population for eliminating malignant cells [173]. This means that CSCs are in desperate need of tailoring ways for T cell suppression. Reportedly, tumor-derived EVs transfer active TGF-β type II receptors to recipient cells. The elicited TGF-β signaling in these groups of cells induces the EMT process, hence promoting a hospitable environment for CSCs, and the stem-like features in low-grade tumor cells are amplified. Meanwhile, the delivery of such tumor-derived EVs to CD8^+^ T cells sets SMAD3 and TCF1 transcription factors in motion, consequently leading to the exhaustion of CD8^+^ T cells and minimizing their anti-tumor function [174, 175]. CSCs are not confined to the production of EVs for the immune system. Through the B7-H1 and galectin-3 pathways, CSCs induce apoptosis in CD8^+^ T cells. It has been revealed that in the lymph nodes of patients suffering from metastatic breast cancer, higher levels of CSCs were detectable, in comparison to patients with less invasive breast cancer. Additionally, studies have demonstrated a significant correlation between the number of recruited regulatory T cells and the levels of CSCs [176]. Along with the activation of regulatory T cells and ceasing the expression of specific tumor-associated antigens (TAAs) and immunosuppressive cytokines, this represents another feasible solution for CSCs to evade immune activation [177].

Normally, CD8^+^ T cells give rise to EVs, which are particularly helpful in terminating malignant cells. To achieve this goal, they tend to put their cargo miR-298–5p, which has been reported to reduce the invasion at cancer sites [178]. According to Zhou et al., granzyme A can also terminate malignant cells by inducing pyroptosis [179]. In return, the EVs that stem from cancer cells seem to be completely different in terms of action. They contain large amounts of TGFβ-1, which, to our knowledge, is responsible for ceasing the immune activity in CD8^+^ T cells [180]. High levels of PD-L1 are also another way for cancer cells to halt the anti-tumor activity in CD8^+^ T cells through the mediating role of EVs [181, 182].

### B lymphocytes

Although a large number of studies have aimed at the critical roles of immune cells in TME and CSC niche formation [183–185], B cells have been somehow neglected in the present literature. However, B cells can efficiently contribute to the anti-tumor immune response through two courses of action: first, via the humoral immune response and second, by presenting recognizable antigens to T cells, along with DCs and other antigen-presenting cells (APCs) [186].

Considering EVs, we have those produced by B cells themselves in comparison to the ones stemming from cancer cells. The EVs that originally originated from B cells contain MHC-I and MHC-II molecules, which are crucially important for APC and their activation [181, 187]. The increased levels of CD39, CD73, and adenosine are also another tool that aids in suppressing the anti-tumor activity in other immune cells [186]. Tumor-derived EVs interfere with B-cell function and, as a result, facilitate immune evasion. These EVs competitively bind to anti-tumor antibodies produced by B cells. Moreover, the EVs intervene in phagocytic and antibody-dependent cytotoxic systems [188]. Increased cargo of PD-1 and PD-L1 in cancer-derived EVs is one mechanism through which the cancer subpopulation weakens the immune system and invades this natural defense system [189]. The presence of CD20 and ABCA3 molecules in these EVs interferes with the biological function of antibodies and inhibits their effect [188, 190].

### TAMs

**FIGURE 4:**
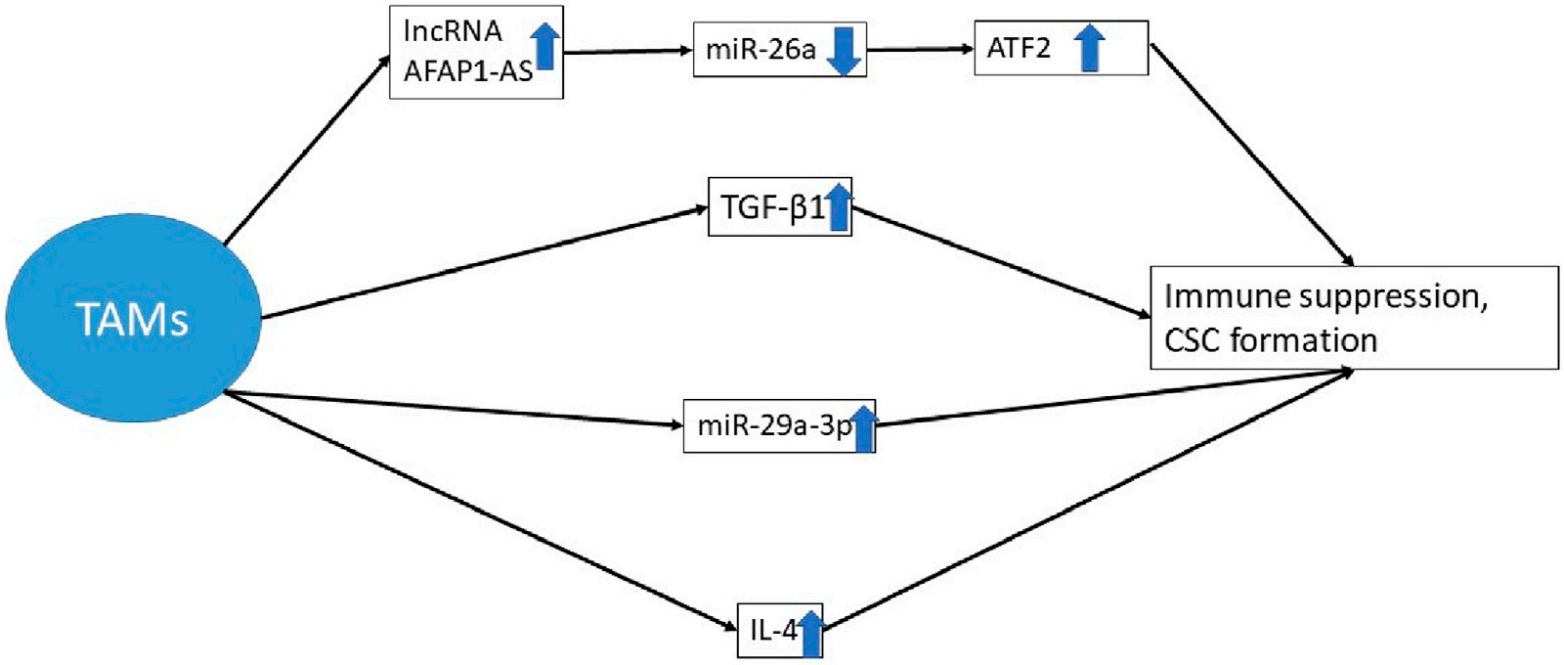
The augmented production of TGF-β1 by TAMs, the enhanced production of interleukin 4, and the overexpression of miR-29a-3p obstruct anti-tumor immune responses. Meanwhile, these exosomes may contain lncRNA AFAP1-AS, which leads to a reduction in the expression of miR-26a. Consequently, there is a modification in gene expression that causes the upregulation of ATF2 [192–196]. * TAM, tumor-associated macrophages; lncRNA, long non-coding RNA; miRNA, micro RNA; CSC, cancer stem cell; ATF 2, activating transcription factor 2.

In physiologic circumstances, the high levels of NF-κB and IL-1β in macrophage-derived EVs are key players in summoning and differentiating T cells and B cells as the main actors in the defense system [188, 191]. Even so, the EVs stemming from cancer cells are not the same when it comes to cargo or function. The higher production of TGF-β1 by TAMs yields a promoted EMT and CSC niche foundation [192]. TAMs preserve the CSC niche through their constant production of cytokines, growth factors, and exosomes. These exosomes may be accommodating lncRNA AFAP1-AS, resulting in downregulation of microRNA-26a (miR-26a). Meanwhile, the alteration in gene expressions yields an elevation of activating transcription factor 2 (ATF2). The ultimate aftermath of it all is the escalated capacity for tumor progression, invasion, and metastasis in CSC niches in esophageal cancer patients [193, 194]. Apart from the described study, another tangible example of triggered cell proliferation and immune escape is the overexpression of miR-29a-3p in ovarian cancer patients [195]. An increased production of IL-4, which halts anti-tumor immune responses, further explains this phenomenon [196]. Ultimately, there seems to be more than one practical way of influencing the immune system through one particular type of cell (Figure 4).

### TANs

Polymorphonuclear myeloid-derived suppressor cells (PMN-MDSCs) have manifested their role in CSC survival as being significant. They have been able to do so through the S100A9-positive exosome [53, 197]. Melanoma-derived EVs have been thoroughly studied in terms of their impact on TANs. Studies suggest that they trigger the production of TAN-N2, the pro-tumor phenotype of PMN. The newly formed TANs are not as efficient in the field of immune responses as they used to be. In other words, they are not as functional in terms of phagocytic and cytotoxic mechanisms, their oxide nitric and peroxynitrite production is relatively impaired, and their extracellular traps are poorly structured. These alterations in the properties of TANs are the bases of an impaired immune response [198]. Moreover, EVs prompt lymph node endothelial cells to produce CXCL8/2, thereby facilitating metastasis [199]. Meanwhile, tumor-derived EVs induce overproduction of PD-L1 in TANs. The ultimate result of this modification is T-cell suppression caused by PD-L-1-rich TANs [200].

### NK cells

NK cells are somewhat responsible for destroying malignant cells. One mechanism by which for these cell groups act is by producing EVs. These physiologically functioning EVs contain tumor necrosis-alpha (TNF-α) and fas-ligand (FasL). As a result, they tend to induce apoptosis in the targeted cancer cell line. Researchers have thoroughly studied this effect in melanoma tissue samples [201]. Meanwhile, these EVs are known to contain cytotoxic proteins such as granzyme A, which enhance the cytotoxic implications of NK cell-derived EVs on their cancer cell lines [202]. IL-15 is yet another EV cargo that aids in the elimination of malignant cells in cancer tissues [203]. In turn, cancer-derived EVs seem to depict different functional targets. Increased levels of TGF-β1 in these macrovesicles are partly to blame for the suppressed immune response in NK cells in malignant tissue [204]. Meanwhile, these cancer-derived EVs are covered with NKG2D ligands, which can greatly decrease the functional viability of NK cells in terms of destroying cancer cells [205]. This action is enhanced more by miR-23a, which is encompassed in these EVs and tends to induce cytolysis in malignant cell lines [182, 206].

CSCs have profound impacts on NK cells. The intercellular interaction between CSCs and NK cells diminishes the presentation of CD71 and CD98. Along with the attenuated glucose uptake, NK cell function against pancreatic cancer cells gets impaired. Immunosuppression is further facilitated by Smad2/3 phosphorylation and elevated TGF-β1 production [207, 208]. The tolerance that is rooted in tumor-derived EVs is reversible in the case of NK cells. The EVs, which have been directly produced by NK cells, can contribute to a better immune response against malignant cells [209].

### MDSCs

The intercellular connection between CSCs and MDSCs is valid in both ways [210]. MDSCs affect stem cell-like features of malignant cells through an elevated level of PGE2. It also yields an over-presentation of PDL-1 factor in ovarian cancer cells [211]. Additionally, MDSCs are capable of inducing the phosphorylation of STAT3 and NOTCH activation subsequently. The ultimate outcome of all mechanisms is an empowered CSC that is readily functional in terms of tumor progression and metastasis. Breast cancer tissue investigations have helped shed light on the matter [212]. CSCs have in turn been taking action on MDSCs. One way these cell groups achieve this is by secreting MIF. This chemical mediator, in turn, induces immune suppression in the TME. Apart from the recent information being uncovered from a precise investigation on glioblastoma tumors [213], cervical cancer has also been assessed for further clarification of CSC and MDSC interactions. The production of PGE 2 on behalf of cervical cancer CSCs leads to diminished immune system function in the TME [214]. EVs derived from malignant tumors, such as melanoma, are also deteriorating the anti-tumor state. Through a boosted level of PD-L 1 expression on myeloid cells, T-cell suppression is the final outcome of such endeavors [215]. The augmented immune suppression capability of MDSCs is a direct repercussion of tumor-derived EVs. Meanwhile, other subsets of myeloid cells, such as DCs, monocytes, macrophages, and granulocytes, acquire pro-tumorigenic properties [216]. A rather general impression on the immune system is further imposed by CSCs. These cells tend to particularly stimulate the production of cytokines, including TGF-*β*, IL-10, IL-4, and IL-13. All components of the immune system are profoundly influenced by their existence. NK cells, T cells, and APCs will be presenting diminished levels of activity [217, 218]. Moreover, an elevated level of TGF-*β1* and MIC-1 cytokines specifically recruit macrophage cells. These cells will respond to CSC products by suppressing routine anti-tumor immune mechanisms [219]. A thorough comprehension of the intercommunication between malignant tissues and the immune system provides an opportunity for designating proper immunotherapy weapons. Cytotoxic T lymphocytes are one of the probable candidates for destroying CSCs. It has been manifested that they are capable of identifying CSC colons both *in vitro* and *in vivo*. Therefore, the Cep55/c10orf3_193 (10) peptide-based cancer vaccine is declared to be an efficient immunotherapy for defeating CSC colons in chemotherapy-resistant colon cancer patients [220–222]. Furthermore, the implementation of T cell-derived EVs and CAR-T cells has also been thoroughly discussed in terms of immunotherapy [223, 224]. Other realms of possibility are being closely investigated. There has been growing evidence of the existence of particular T cells that solely focus on [225]. Another promising immunotherapy mechanism that has been thoroughly investigated is the effect of IFN-*γ*-treatment on CSCs. Apparently, CSCs tend to depict higher levels of vulnerability to T cell-mediated immune mechanisms in the presence of IFN-*γ* [217, 218]. CSCs are inherently resistant to T-cell immune responses [226]. This phenomenon may be rooted in the diminished MHC class I presentation in these cell groups [227]. Although a lack of NK cell-activating ligands may be the case in some cancers, other tumors are enclosed by diverse ligands for NK cells (including the poliovirus receptor). Thus, it is interpretable that NK cells may be favorable tools for defeating these classes of malignancies [227]. NK cells are among the most powerful rivals of CSCs. A remarkable number of studies have delved into their potential as immunologic therapeutic agents in recent years. MiR20a–MICA/MICB is a possible pathway for eliminating breast CSCs [228]. CD24^+^/CD44^+^, CD133^+^, and aldehyde dehydrogenase are the CSC markers that are efficiently identifiable by NK cells. In return, NK activation ligands MICA/B, Fas, and DR5 are more frequently presented on CSCs [229–231]. A number of recognizable instances of matter can be found in breast cancer [231] and melanoma [232]. Not only are NK cells *per se* influential in treating cancer, but the NK cell-derived EVs can also be proven worthy of attention in fields of immunotherapy [233]. Immunotherapy agents have advanced over time, and a more profound understanding of the regular immune regulations at the TME can be an incredible assistant to the results.

### CAFs

CSC-derived exosomes consist of microRNA and protein molecules, which could be responsible for the transformation of stromal cells and the initial formation of CAFs. These newly formed cells present enhanced proliferation, migration, and secretory characteristics. They will be increasingly secreting cytokines and assisting in the foundation of a TME launch [234]. The secretion of the hedgehog ligand SHH by CSCs induces paracrine effects on CAFs. In return, CAFs provoke stem cell-like properties among CSCs. Vismodegib is known for its capability of inhibiting Hedgehog signaling pathways. This therapeutic agent has been evaluated for use in breast cancer and has offered promising results [235]. This signaling pathway is not the only feasible way of interaction between CAFs and CSCs so far. The encompassed molecules in cancer-derived EVs (such as miRNAs, proteins, lncRNAs, and mRNAs) augment the characteristics of CAFs, along with other proposed mechanisms [236]. It has been proposed that a facilitated EMT and angiogenic process is in motion to provoke metastasis through EV transfer between CAFs and CSCs [237].

### Pericytes

The pericyte evolution and transformation are initially a consequence of hypoxia-derived EVs encompassing TGF-β1 mediators. Targeting this particular pathway using therapeutic agents such as ibrutinib will indeed lead to more efficient glioblastoma eradication. Its anti-tumor function is further enhanced as bevacizumab is combined with ibrutinib [238]. Pericytes are subsequently responsible for angiogenesis and metastasis promotion [239]. Glioblastoma CSC-derived pericytes have been specifically targeted. Based on the ultimate outcome, pericyte inhibition is a potent anti-tumor remedy [240]. The role of pericytes in the induction of metastasis is yet to be fully comprehended. A limited number of studies have delved into the matter. It has been proposed that in a case of lung adenocarcinoma with distant brain metastasis, CD44^+^ CSCs have given rise to specific pericytes. These cells employed the G-protein-coupled receptor 124 (GPR124)-enhanced trans-endothelial migration (TEM) pathway to facilitate their migration into the blood vessels. They are well-capable of self-preservation in the bloodstream, and further on, they managed to successfully extravasate from the vessels into the desired location of metastasis. This mechanism appears to be a promising potential site of therapy for metastasis control [241].

### Adipocytes

Breast cancer has introduced opportunities for the examination of the role of adipocytes in cancer pathogenesis. The dominant mechanism, based on which the critical role of adipocytes in tumor progression and metastasis is described, is the induction of stem cell-like properties. IL-6 and leptin signal CSCs and enhance stem cell-like features among them [242, 243]. Furthermore, adipocytes are reportedly competent to promote the activation of STAT3 and, thereafter, the inhibition of miR-200a, as well as the elevation of ZEB2 expression. The aftermath of this cascade of events is that colorectal CSCs acquire metastatic phenotypes [244]. Further investigation into breast cancer [245, 246] and prostate cancer [247] is being carried out to aid in clarifying the enigmatic pathological pathways.

### Endothelial cells

Endothelial cells are, similar to other TME components, apt to reinforce stem cell-like and self-renewal features in head and neck CSCs [248]. The proposed mechanism for the so-called consequence is through the production of basic fibroblast growth factors by tumor microvascular endothelial cells. This mechanism was uncovered by Fessler et al. while examining glioblastoma cancers [249]. Colorectal malignancies have also been evaluated by Lu et al., and comparable results were acquired [250]. Meanwhile, endothelial cells are undoubtedly among the most influential cells in the angiogenesis process. Epithelial ovarian cancer-derived EVs, which reportedly contained miR-141-3p, promote angiogenesis properties in these groups of cells [251].

### ECM remodeling

EVs encompass nucleic acids and proteins, which may include diverse types of enzymes. Matrix metalloproteinases, heparanases, hyaluronidases, and aggrecanases are all examples of existing enzymes in cancer-related EVs. This collection of enzymes is also acknowledged as matrix-remodeling enzymes. As one may interpret from the associated name, these enzymes tend to disrupt the structure of the former ECM at the tumor site. Furthermore, enzyme regulators, including extracellular matrix metalloproteinases, inducers, and tissue inhibitors of metalloproteinases, are simultaneously transferred via EVs and aid in regulating ECM remodeling and the activities of EV-associated matrix-remodeling [252]. A closer investigation into thyroid cancers reveals that these EVs initially originated from CAFs. Following their release, they provide degrading enzymes and required regulators. Particularly, matrix metalloproteinase 2 is known to be a vital enzyme for the degradation of the ECM. Tumor invasion is the clinically tangible outcome of the process [253]. TAMs are also manifested as cooperating in the ECM remodeling process. Alongside producing EVs with degrading profiles, these cells tend to stimulate VEGF secretion and angiogenesis. Meanwhile, an elevated level of proteinase, which directly originates from TAMs, also intensifies the degrading properties of the complex. Inducing inflammatory responses is another suitable mechanism to facilitate ECM remodeling during tumor progression and invasion [254].

### Tumor angiogenesis

**FIGURE 5:**
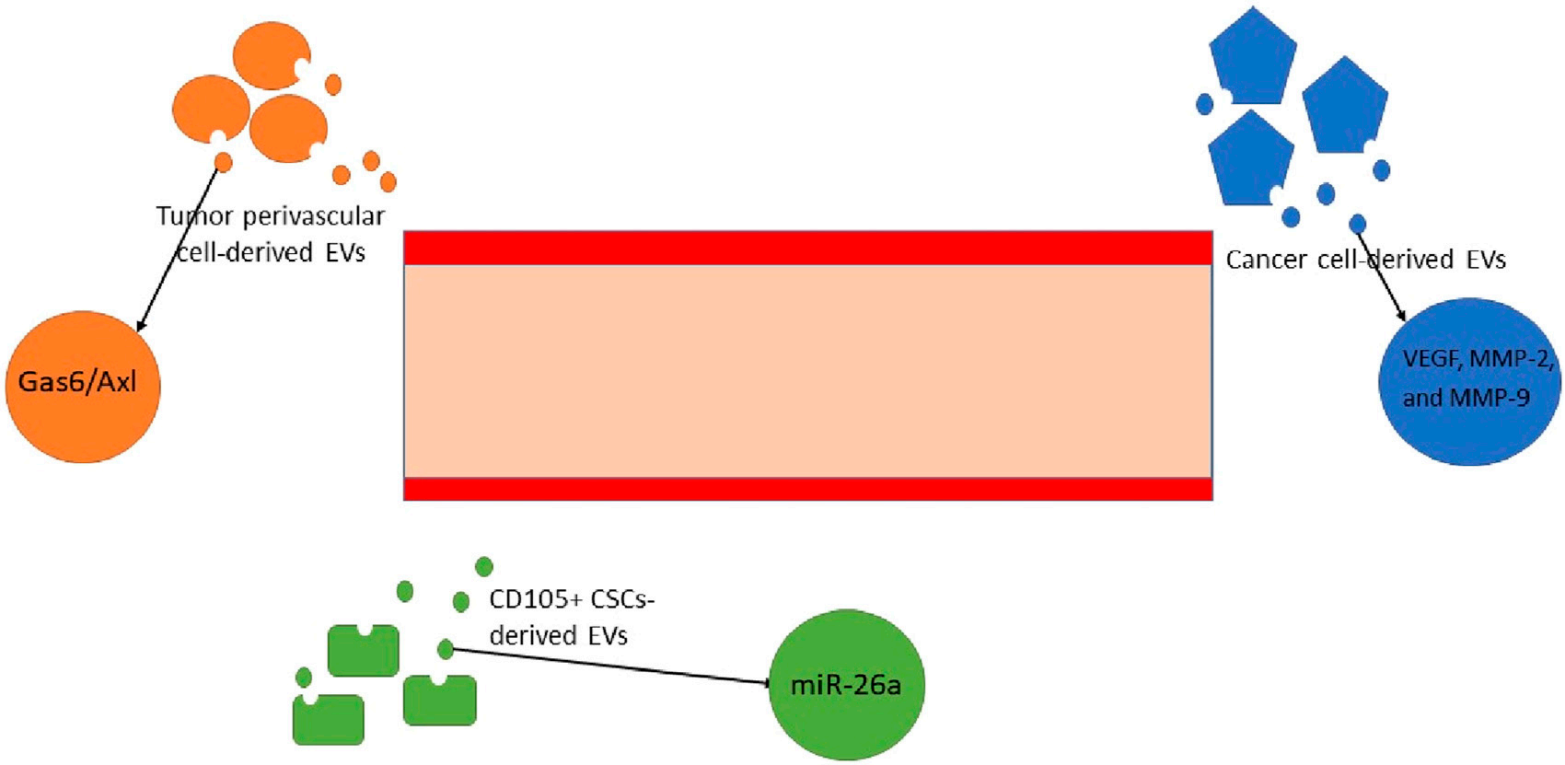
Angiogenesis is directly provoked by multiple mechanisms, all of which heavily rely on the crucial role of EVs. The exosomes that contain miRNA-26, VEGF, MMP-2, and MMP-9 and stimulate the Gas6/Axl signaling pathway are responsible for the growth of micro-vessels in tumor and malignant sites [92, 255–257]. * EV, extracellular vesicle; VEGF, vascular endothelial growth factor; MMP, matrix metalloproteinase.

Angiogenesis is a critical factor to ensure tumor progression and metastasis. All cells rely on the micronutrients, which are generally transferred by blood vessels. Cancer cells are specifically dependent on this blood flow due to their enhanced proliferation and activity. Tumor perivascular cell-derived EVs employ the Gas6/Axl axis to provoke angiogenesis [255]. Over and above, CSCs release EVs containing miR-26a. This nucleic acid molecule has been demonstrated to promote endothelial cells to embark on angiogenesis. These data were recently extracted through the assessment of glioblastoma malignancies in humans. There is also proof that VEGF, MMP-2, and MMP-9 are critical players in angiogenesis. They are accessible through glioma cancer-derived EVs [256]. The glioblastoma cancer site has been harbored by other subsets of cancer-derived EVs. These exosomes contain VEGF A mediators, which are instrumental factors in both the induction of angiogenesis and increasing vascular permeability [257]. To accentuate the role of VEGF, further investigation into glioma and glioblastoma malignancies was carried out. Hence, the miR-21/VEGF/VEGFR2 signaling pathway has been identified as another influential ring of the chain [92]. In addition to brain tumors, a number of studies have been conducted to confirm the existence of similar pathways in renal cell carcinomas. They have been affirmative of the existence of CD105+ CSCs and their derivative EVs, which provoke angiogenesis [94, 258]. Angiogenesis holds other components of the TME and CSC niche together and provides nutritional support throughout tumor progression, invasion and metastasis (Figure 5).

## Clinical implications and translation

Recently, with regard to CSC properties, it seems that more studies on these cells will open a new horizon in cancer research and help resolve the cancer therapeutic dilemma.

### Potential as diagnostic and prognostic biomarkers

**FIGURE 6:**
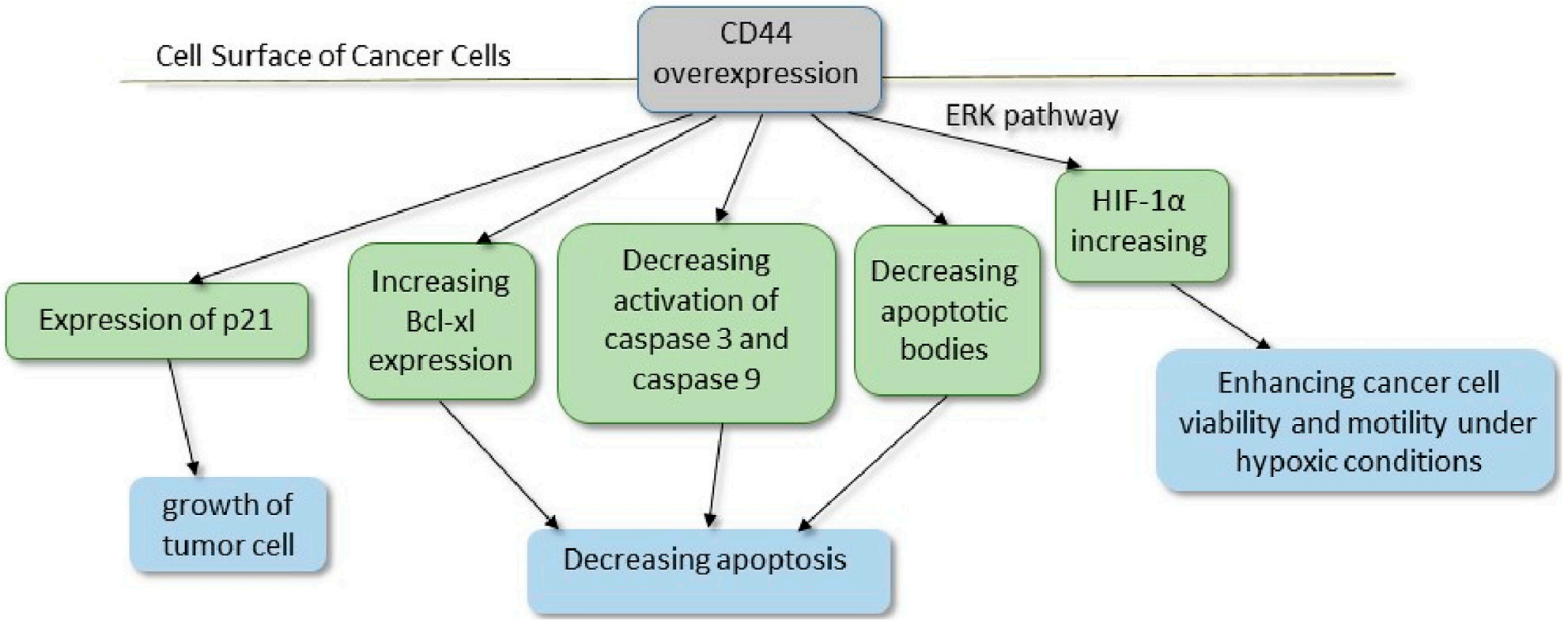
CD44, a cell surface receptor for hyaluronic acid (HA), facilitates both cell–cell and cell–matrix interactions. Furthermore, upregulated active caspases 3 and 9 in CD44-deficient tumor cells have shown the involvement of the mitochondrial pathway in apoptosis. Additionally, studies have considered that the ratio of anti-apoptotic Bcl-xl to pro-apoptotic Bak was shifted toward apoptosis in CD44-deficient tumor cells due to decreased Bcl-xl expression. P21, a cell cycle regulator, is upregulated in CD44^+^ cells, which seems to be necessary in tumor cell growth [289–291]. * ERK, extracellular signal-regulated kinase; HIF, hypoxia-inducible factor.

Routinely, various strategies and serum markers are applied to different cancer diagnoses to determine their prognosis. Recently, CSCs and their special properties have been used as promising biomarkers to improve early diagnosis and effective treatments. Additionally, conventional methods of staging and grading for most cancers are not effective enough in estimating the prognosis. Accordingly, CSC biomarkers are suggested for use in estimating the cancer’s clinical behavior and survival outcomes. CSCs, as a group of malignant cell populations in a tumor environment, have selective characteristics and properties. Accordingly, CSCs are drug-resistant and relatively slow in growth. Hence, they are supposed to play a key role in tumor resistance to anti-cancer therapeutics that lead to poor prognosis. Additionally, metastasis, another challenge in cancer management, is related to CSCs. In this regard, it seems that to achieve an astonishingly early and effective cancer eradication, we should target CSCs [259]. Targeting CSCs requires the identification of specific markers for CSCs in each tumor. Various cell surface markers are used to define CSCs, including CD24, CD34, CD44, CD133, CD139, CD166, and ESA (Table 4) [285]. Thomsen–Friedenreich (TF) antigen, as an oncofetal antigen, is overexpressed at high levels in malignant tissues. A body of literature introduced TF antigen as a highly tumor-specific CSC marker [286, 287]. In this regard, CD24 and CD44 are identified as cell markers on the surface of CSCs in most types of tumors. However, further studies are required to prove them to be common markers in CSCs [288] (Figure 6). A study surveyed the CSCs–EVs in CCRCC patients with lung metastasis and found that CD103+ EVs were detected at high levels in the blood samples of these patients. They have demonstrated that CD103+ guides EVs to target cells and organs, which facilitates metastasis. Hence, CD103+ EVs in CCRCC patients could be used as a prognostic and even diagnostic biomarker [79]. As discussed in previous paragraphs, micro-RNA and lncRNA are considered promising clinical targets for cancer. Both specific dysregulated (up or downregulated) miRNAs and lncRNAs are found in special tissues and even in other biological samples (e.g., blood, plasma, serum, urine, exosomes, and stool), which leads to considering them as future biomarkers. For instance, the literature has suggested some of the exosomal miRNAs, including miR-21 and miR-210F, as diagnostic biomarkers for pancreatic cancer [292]. Focusing on the isolation and characterization of CSCs will be helpful in better understanding tumor formation, enabling early diagnosis, and developing more effective novel anti-cancer treatments to target this cell population.

**TABLE 4:** Cancer stem cell surface markers in various cancers. This table illustrates some examples of CSC surface markers that are studied in the literature in different cancers. These cell markers could be used not only as a marker to isolate CSCs but also as diagnostic and prognostic factors.

Cancer	Potential cell marker	Reference
Breast cancer	CD14, CD24, CD29, CD44, CD49f, CD90, and ALDH1	[260–263]
Colorectal cancer	CD24, CD44, and EpCAM^a^	[264–266]
Squamous cell carcinoma (SCC)	CD29, CD44, and ABCG2	[267, 268]
Prostate cancer	CD44, α2β1 integrin, CD133, CD49f, and EphA2^a^	[269, 270]
Pancreatic cancer	CD24, CD44, ESA, CD133, c-Met, and ALDH^a^	[69, 271, 272]
Ovarian cancer	CD24, CD44, CD133, CD90, and SSEA	[273, 274]
Uterine leiomyoma	CD34 and CD49b	[275]
Renal cell carcinoma	CD44 and CD105	[276–278]
Non-small cell lung cancer	CD44, CD166, and EpCAM^a^	[279, 280]
Gliomas	CD90	[281]
Lipomas	CD34	[282]
Hepatocellular carcinoma	CD34 and CD133	[283, 284]

^a^ EpCAM, epithelial cellular adhesion molecule; EphA2, ephrin type-A receptor 2; ALDH, aldehyde dehydrogenases.

### Therapeutic targeting strategies utilizing extracellular vesicles and cancer stem cells

Invasiveness and metastasis of tumor cells are attributed to tumor stromal cells, including MSCs, CAFs, macrophages, and myeloid-derived suppressor cells. Tumor stroma with various EVs and factors, such as TGF-β, leads to the malignant transition of tumor cells to mesenchymal tumor cells, which are mainly responsible for aggressiveness. Targeting tumor stroma, especially MSCs, will be effective in cancer therapy. As discussed in previous paragraphs, CSCs use various mechanisms to confer resistance to cancer treatments. In this regard, CD44, especially variant isoforms (CD44v), is implicated as one of the CSC markers. As a consequence, CD44v protects CSCs from stress induced by ROS by promoting the cellular anti-oxidant. Therefore, the therapeutic CD44v system may be helpful in enhancing apoptosis in tumorigenesis cells [293]. Moreover, CD44 also acts as a recyclable receptor for hyaluronic acid (HA). CD44 overexpresses in CSCs and promotes epithelial–mesenchymal transition. Meanwhile, the expression of P-glycoprotein (P-gp), a multidrug resistance 1 (MDR1) protein, requires HA. In addition, the interaction of HA and CD44 enhances epidermal growth factor receptor (EGFR)-mediated pathways, which leads to tumor progression. Altogether, CD44 is considered a factor in drug resistance and invasion in cancers. Monoclonal antibodies against different CD44 variant isoforms have recently been taken into account in cancer therapies (Figure 3) [294]. CAFs, as a group of non-immune cells in a tumor environment, are identified in various tumors. In healthy tissues, normal fibroblasts and myofibroblasts contribute to tumor-suppression. However, heterogeneous CAFs play a pivotal role in cancer progression. It is hypothesized that CSCs, like other stem cells, require a special environment and supportive niche to expand and maintain their stemness characteristics. CAFs, through providing this niche for CSCs, have recently become a hot topic in cancer therapy research. A CAF subset with high CD10 and GPR77 expression is identified in breast and lung cancer. CD10+GPR77+ CAFs through the NF-κB pathway correlate with survival niches for CSCs, tumor formation, and chemoresistance. Hence, targeting CAFs in tumors, including anti-GPR77 antibodies, could help in promoting chemoresistance and prognosis (Figure 3) [100, 139]. EVs with unique characteristics, including the ability to cross biological barriers, stability, and carrying various materials, are considered promising therapeutics (Table 5). Kazemi et al. assessed EVs derived from CSCs in breast cancer. CSC-derived EVs could deliver LNA-anti-miR-142-3p to breast cancer CSCs. LNA-anti-miR-142-3p reduced tumorigenicity by inhibiting miR-142-3p and miR-150 [302]. However, there are various concerns about utilizing CSCs and their derivatives, especially in cancer patients. Hence, further clinical studies in this area are required. EVs are secreted from any type of cell, including bacteria. Recently, research on the application of bacteria-derived EVs has become a hot topic. Behzadi et al. purified EVs derived from *Lactobacillus rhamnosus GG* (LDEVs) and tested them on a hepatic cancer cell line. These EVs increased the bax/bcl2 expression ratio as an apoptotic index. Consequently, LDEVs induce apoptosis in hepatic cancer cells [136]. Furthermore, EVs of *Lacticaseibacillus paracasei PC-H1* (LpEVs) could interact with colon cancer cells. LpEVs stimulate the PDK1/AKT/Bcl-2 signaling pathway as a trigger for apoptosis. Hence, LpEVs could eliminate colon cancer cells by inhibiting proliferation, migration, and invasion [303]. Moreover, EVs derived from bacteria, in addition to other bioengineered EVs and chemical treatments, open up new opportunities for the treatment of various cancers. As discussed in previous paragraphs and subtitles, most of the studies conducted are *in vitro* and *in vivo*, and clinical studies in this field are limited. Furthermore, moral concerns and potential problems with stem cells, the application of CSCs, and their products pose a challenge. In this regard, there are various gaps between pre-clinical and clinical research. So comprehensive interdisciplinary studies necessitate the application of novel techniques in diagnosing, treating, and prognosing different cancers.

**TABLE 5:** Some of the clinical trials with details are illustrated.

Cancer type	Phase	Outcome measure	Primary outcome	Status	Country	Reference
Retinoblastoma	Phase I	Diagnostic	Detection of the type of tumors in RB1-mutation carriers	Completed	France, Germany, and Netherlands	NCT04164134
Prostate cancer	Phase I and II	Prognostic	Validation of the ability of those candidate exosomal microRNAs in differentiating pathological insignificant and significant prostate cancer	Completed	Hong Kong	NCT03911999
Melanoma	Phase I	Therapeutics	Feasibility and safety of the autologous exosomes pulsed with MAGE 3 peptides for the immunization of stage III/IV melanoma	Completed	France	[295]
Non-small cell lung cancer	Phase I	Therapeutics	Safety, feasibility, and efficacy of autologous Dex loaded with the MAGE tumor antigens	Completed	USA	[296]
Non-small cell lung cancer	Phase II	Therapeutics	Benefit of IFN-γ-Dex loaded with MHC class I- and class II-restricted cancer antigens	Completed	France	[297]
End-stage lung cancer	Phase I	Therapeutics	Reversing drug resistance of tumor-repopulating cells using tumor cell-derived microparticles (T-MPs) containing anti-tumor drugs (cisplatin)	Completed	China	[298]
Malignant pleural effusion	Randomized parallel controlled trial	Therapeutics	Immunotherapeutic effect of methotrexate (MTX)-packaging tumor cell-derived microparticles (MTX-MP)	Completed	China	[299]
Colorectal cancer	Phase I	Therapeutics	Immunotherapeutic effect of the ascites-derived exosomes in combination with the granulocyte-macrophage colony-stimulating factor (GM-CSF)	Completed	China	[300]
Colon cancer	Phase I	Therapeutics	Immunotherapeutic effect of the curcumin-loaded exosomes and assaying the concentration of curcumin in normal and cancerous tissue	Recruiting	USA	NCT01294072
Metastatic pancreas cancer	Phase I	Therapeutics	Immunotherapeutic effect of KRASG12D siRNA-loaded exosomes	Active	USA	NCT03608631
Obstructive extrahepatic cholangiocarcinoma	Phase I	Therapeutics	Immunotherapeutic effect of the methotrexate-containing plasma-membrane microvesicles derived from apoptotic human tumor cells	Completed	China	[301]

### Challenges and future directions for clinical translation

Interestingly, some studies have recently claimed that routine chemotherapy and radiotherapy could increase the risk of tumor progression and metastasis. Accordingly, a study has proposed a breast cancer model. Systemic standard chemotherapy in this model leads to an inflammatory and catabolic microenvironment that induces stemness in adjacent tissues and promotes tumorigenesis [304]. In this regard, novel techniques are required in this area. Targeting and eradicating the CSC population seems like a novel insight taken into account recently in tumor therapeutics. Recently, fasudil, approved for clinical use in vascular pathologies, has been suggested for application in cancer therapy. Fasudil is a Rho-associated protein kinase (ROCK) inhibitor. Guerra et al. reported that fasudil, with its role in inhibiting cell migration, is considered a novel prophylaxis for cancer metastasis [305]. Furthermore, glioma resistance to chemotherapy has been attributed to ROCK2 activity. Hence, *in vivo* and *in vitro* investigations have demonstrated that fasudil could increase chemosensitivity in resistant gliomas [306]. However, further studies are needed to prove this hypothesis. As discussed previously, *P*-gp is responsible for multidrug resistance. Paclitaxel (PTX) is a conventional, effective chemotherapy for various cancers. However, in the body, it is considered a substrate for P-gp. Salinomycin (SLM), targeting breast cancer stem-like cells, can act as a P-gp inhibitor. Furthermore, overcoming apoptosis resistance and dysregulating the Wnt signaling pathway makes it a good candidate for promising combination therapy. Hence, it seems that combination therapy with PTX and SLM could overcome chemo-resistance and achieve effective CSC eradication. In this regard, conventional chemotherapy and radiation therapies should be replaced with novel combinational therapeutics [294]. Angiogenesis and neovascularization are other factors that participate in tumor growth and survival. The transition of tumor-infiltrating MSCs to myofibroblasts by neovasculature development-related cytokines promotes angiogenesis. In this regard, GW4869, a noncompetitive inhibitor of sphingomyelinase (SMase), is known to reduce exosome generation and release. Likewise, GW4869 could reduce inflammatory cytokines such as interleukin (IL)-1β, IL-6, and TNF-α in macrophages. GW4869-treated CD8^+^ T cells could control angiogenesis by reducing cytokines [132]. In addition, macrophage polarization is another target of anti-cancer treatments. In this regard, Peng et al. investigated the GW4869 effect on prostate cancer. In prostate tumor environments, EVs modulate the M2 transition through the AKT and STAT3 signaling pathways. GW4869 could inhibit the release of EVs, which leads to the termination of differentiation into M2 cells and tumor progression [141]. Additionally, some of the recruiting and completed clinical trials are described in Table 4. Altogether, it is hypothesized that EVs have immune suppression effects, although these EVs contain various contents that not only suppress immunity but also could suppress the tumor, conversely [307]. Hence, EVs are a double-edged sword, and they could be effective in tumor suppression in some cancers. Additionally, unfortunately, most of the studies in this field are *in vitro* and *in vivo*, with limited human studies. Altogether, further studies, particularly human studies, are required to determine the exact pathways, target genes, and molecules needed to attain the most effective achievements.

## Conclusion

Tumor progression, invasion, and metastasis are mechanisms that are vital to decode. Interfering with these mechanisms yields enhanced anti-tumor therapy and metastasis prevention, a goal that has been pursued for a considerably long period of time by oncologists. Recently, scientific evidence has drawn scientists’ attention to the unnegotiable and crucial role of EVs and CSCs in cancer pathology [155, 308]. Not only are they manipulating the normal physiological routines of cell cycles individually, but they are also contributing to one another in numerous complicated ways [13]. Although they are yet to be fully comprehended, tumor heterogeneity relies heavily on CSCs and EVs. The formation of tumor cell colonies and the foundation and evolution of diverse cell subpopulations are the keys to preserving tumor heterogeneity [309, 310]. A number of probable signaling pathways have been introduced to explain the matter at hand. Signaling pathways undoubtedly provoke the formation, progression, invasion, and metastasis of tumors [311]. The adverse effects of CSCs and EVs do not end here. Tumor resistance to medical treatments has been an unnerving challenge for healthcare providers in the oncology field. Therapy resistance has also been traced back to these two cellular components [312, 313]. Furthermore, cancer relapse and recurrence have reportedly been another outcome of this cooperation [314]. In cancer therapy, CSCs and EVs can be suitable targets for chemical agents due to their undeniably significant role in tumorigenesis [315–317]. This therapeutic approach has been put into trial in a number of cancers (colorectal cancer [318], testicular cancer [319], breast cancer [320], ovarian cancer [321], and lung cancer [154]). Although these clinical trials have been limited in number, not all of them were carried out with an acceptable sample size. It is noteworthy that our limited understanding of the way CSCs and EVs function has led to a weakness in drug designation. To make matters worse, we lack adequate engineering techniques and tools for isolating or targeting these cell subpopulations. Clinically inapplicable diagnostic and therapeutic approaches can be a result of the recently discussed matters. In the present study, the intercellular communications between TME cells, CSCs, and EVs have been thoroughly discussed. Consequently, a considerable number of responsible pathways have been identified for tumor progression, ECM remodeling, and angiogenesis [322]. As mentioned, oncologists have highlighted the impressive role of CSCs and EVs in tumor pathology as precious targets for therapeutic approaches [315, 323]. These novel treatments, along with other innovative alternatives such as gene therapy [324], immunomodulatory agents [325], and artificial intelligence applications [326] [327], are considered valid future possible courses of action. Even so, the currently existing literature is not as helpful as one may hope. We should take the initiative and embark on the pathway of truth discovery. A more transparent overview of the ongoing pathways assists scientists in laying out target-oriented, precise, and novel diagnostic, prognostic, and therapeutic approaches. To our knowledge, there have not been a sufficient number of human clinical trials on the possible implications of CSCs and EVs. This can also be a great field for further exploration in the future. There are undoubtedly various other innovative approaches that could be employed in implementing these two cellular mechanisms in cancer diagnosis, prognosis, and treatment. The ultimate goal stands to be the utilization of our knowledge of CSCs and EVs for assisting scientists in their upcoming challenges in the field of oncology.

## Figures and Tables

**Table:** 

**AML**	Acute myeloid leukemia
**CSCs**	Cancer stem cells
**EVs**	Extracellular vesicles
**P-gp**	P-glycoprotein
**lncRNAs**	Long non-coding RNAs
**PDAC**	Pancreatic ductal adenocarcinoma
**MIF**	Macrophage migration inhibitory factor
**EMT**	Epithelial–mesenchymal transition
**CCRCC**	Clear cell renal cell carcinoma
**ncRNAs**	Non-coding small RNAs
**MVs**	Micro vesicles
**TF**	Tissue factor
**MDR**	Multidrug resistance
**TME**	Tumor microenvironment
**ECM**	Extracellular matrix
**NK cells**	Natural killer cells
**NKT cells**	Natural killer T cells
**TAMs**	Tumor-associated macrophages
**MDSCs**	Myeloid-derived suppressor cells
**DCs**	Dendritic cells
**TANs**	Tumor-associated neutrophils
**CAFs**	Cancer-associated fibroblasts
**TAAs**	Tumor-associated antigens
**APCs**	Antigen-presenting cells
**miR-26a**	microRNA-26a
**ATF2**	Activating transcription factor 2
**PMN-MDSCs**	Polymorphonuclear myeloid-derived suppressor cells
**TEM**	Trans-endothelial migration
**MSCs**	Mesenchymal stem cells
**CCRCC**	Clear cell renal cell carcinoma
**ROS**	Reactive oxygen species
**HCC**	Hepatocellular carcinoma
**EGFR**	Epidermal growth factor receptor
